# Association between interleukin gene polymorphisms and the risk of pneumoconiosis: a systematic review and meta-analysis

**DOI:** 10.3389/fmed.2025.1479730

**Published:** 2025-03-20

**Authors:** Lu Liu, Xiaowei Tian, Yilin Guo, Yanyan Yu, Yamei Wang, Wenjing Wang, Jun Meng, Guifang Li, Xiaojuan Sun

**Affiliations:** ^1^Department of Occupational Diseases, Weifang People's Hospital, Weifang, Shandong, China; ^2^Department of Neurosurgery, Dongping Xian People's Hospital, Taian, Shandong, China

**Keywords:** interleukin, gene polymorphism, pneumoconiosis, susceptibility, meta-analysis

## Abstract

Numerous studies have demonstrated that interleukin (IL) plays an essential role in the development of chronic inflammatory diseases, especially in pneumoconiosis. The association between various IL gene polymorphisms and pneumoconiosis susceptibility has been investigated extensively, but the results remain controversial. A literature search was conducted using PubMed, EMBASE, Web of Science, Cochrane Library, China National Knowledge Infrastructure (CNKI), and Wanfang database to obtain relevant studies before 22 January 2025. Subsequently, odds ratios (ORs) with 95% confidence intervals (CIs) were used to evaluate the strength of correlations. A sensitivity analysis was performed to evaluate the robustness and reliability of the included studies. Overall, there was a significant association between IL-1RA +2018 and IL-6 -634 with the risk of pneumoconiosis. The IL-1RA +2018 variant was positively associated with an increased risk of pneumoconiosis among both Asians and Caucasians. In contrast, the IL-6 -634 genotype was associated with a lower risk of pneumoconiosis among Asians. Additionally, the IL-1RA +2018 genotype was significantly linked to a predisposition to coal workers’ pneumoconiosis (CWP) and silicosis. The IL-6 -634 mutant significantly decreased silicosis and CWP risk. Additional large-scale replication studies are needed to elucidate the precise role of various IL SNPs in the etiology of pneumoconiosis.

## 1 Introduction

Pneumoconiosis is an occupational disease caused by inhaling free crystalline silica particles, which deposit in the interstitial lung parenchyma (1, 2). There are an estimated 527,500 cases of pneumoconiosis worldwide, and the mortality rate remains high, with more than 21,000 deaths being reported every year (3). Regrettably, inadequate monitoring methods in less-developed countries lead to serious underestimations of the exact numbers of silica-exposed workers. This underreporting can result in higher rates of morbidity and mortality than previously reported (4). Pneumoconiosis generally manifests in different forms, such as coal workers’ pneumoconiosis (CWP), silicosis, and asbestosis, depending on the types of dust inhaled. The diagnosis of pneumoconiosis is mainly based on the history of exposure to harmful dust, abnormal chest radiographs, and pulmonary function tests that may show extensive alveolitis, emphysema, and pulmonary fibrosis (5, 6). Some researchers have identified the altered microRNA (miRNA) expressions in human or animal models as feasible biomarkers for the early diagnosis of pneumoconiosis, such as miR-16, miR-21, miR-29a, miR-155, miR-200c, miR-206, and miR-146a (7, 8). Recent advances in the treatment of pneumoconiosis, including the anti-fibrosis medication pirfenidone and mesenchymal stem cell therapy, indicate potential for slowing the disease progression. However, these studies are at in the early stages, and the safety and efficacy of these approaches have not yet been evaluated in clinical settings (9, 10).

Pneumoconiosis occurs due to the accumulation of carbon and silica from inhaled coal dust that activates humoral and cellular immune responses and sensitization in the damaged lungs (11–13). The disease is characterized by chronic inflammation and fibrosis (14). Mechanically, the inhalation of silica and dust-related particles is absorbed by macrophages, thereby activating injured alveolar macrophages to release pro-inflammatory and fibrotic mediators (15, 16). Then, these inflammatory mediators recruit inflammatory cells into the alveolar walls and spaces and further remodel the process through stimulating fibroblast proliferation and collagen synthesis (17, 18). The early stage of pneumoconiosis can be asymptomatic, but the advanced stage generally results in disability and premature death (19). Several factors contribute to the pathogenesis of silicosis, including the concentration, exposure time and frequency of respirable crystalline silica, gene–environment interactions, and individual susceptibility (20–24). Despite having similar exposure histories, not all individuals developed lung fibrosis, indicating that genetic factors in the host may affect the progression of silicosis (25, 26). Currently, genome-wide association studies (GWAS) have identified a strong association between some common single-nucleotide polymorphisms (SNPs), such as small nucleolar RNA host gene 14 (SNHG14), desmoplakin (DSP), and laminin beta 1 (LAMB1), and pneumoconiosis (27, 28).

.Growing evidence has shown that cytokines and their receptor variants play crucial roles in various biological processes, such as inflammation and immune responses, and mediate pathogenic effects in humans exposed to detrimental dusts (29–31). Among these cytokines, the interleukin 1 (IL-1) gene, which encodes the inflammatory cytokines IL-1α and IL-1β and the competitive antagonist, IL-1 receptor antagonist (IL-1RN), is located on chromosome 2q14 and spans nearly 400 kb of genomic DNA (32, 33). IL-1 was highly expressed in silicosis and aggravated pulmonary fibrosis by modulating the synthesis of collagen (34, 35). Moreover, the elevated levels of IL-6 were observed in the lung tissues and serum of pulmonary fibrosis patients (36). Being a phosphorylated glycoprotein with 185 amino acids, IL-6 plays a role in the inflammation, bone metabolism, and C-reactive protein regulation. The human IL-6 gene is localized on chromosomes 7p21-24 and mainly modulates the transcriptional level via regulatory elements in its 5′ flanking region (37, 38). The position of the IL-6 -174 and IL-6 -634 polymorphisms in the promoter region has been widely studied, and these two polymorphisms exhibited a weak linkage disequilibrium (39, 40). In addition, IL-17 is a multifunctional cytokine produced by Th17 cells, which is involved in the pulmonary fibrosis process via recruiting and activating neutrophils and even other cytokines, such as transforming growth factor-β1 (TGF-β1), IL-1β, IL-6, and IL-13 (41, 42). The pivotal members of the IL-17 family are IL-17A and IL-17F, both of which are located on chromosome 6 (6p12) and are positioned very close to each other (43–45).

.A number of studies have evaluated the association between various IL gene SNPs and the risk of pneumoconiosis; however, the findings remain controversial. For example, Yucesoy et al. first proved no significant relationship between IL-1α and IL-1β polymorphisms and the risk of silicosis. Instead, they found a significant correlation with IL-1RA (46). In 2018, Volobaev et al. reported that the IL-1β genotype was significantly associated with the risk of silicosis, but there was no correlation between the IL-6 and IL-12 genotypes (47). It has been reported that the IL-17F variant was remarkably associated with the silicosis risk, and the G allele may have a protective effect (48). Similarly, Hassani et al. discovered a positive correlation between the IL-17F allele and the silicosis risk, while no such correlation was observed with the IL-17A genotype (49). Therefore, we performed this meta-analysis to precisely evaluate the association between the IL-1α +4845G/T, IL-1β +3953C/T, IL-1RA +2018T/C, IL-1β -511C/T, IL-6 -634C/G, IL-6 -174G/C, and IL-17A -832A/G polymorphisms and silicosis susceptibility.

## 2 Materials and methods

### 2.1 Literature search strategy

This meta-analysis was conducted in accordance with the Preferred Reporting Items for Systematic Reviews and Meta-Analyses (PRISMA) guidelines (50). All collected data were based on previously published studies. Therefore, no ethical approval was required. We performed a literature search using PubMed, EMBASE, Web of Science, Cochrane Library, Scopus, Google Scholar, China National Knowledge Infrastructure (CNKI), and Wanfang database for relevant studies published up to 22 January 2025 without any language restrictions. Additional sources were searched in the Cochrane Central Register of Controlled Trials (CENTRAL), National Research Register (NRR), and Clinical Controlled Trials (CCT) to identify unpublished gray literature. The following keywords were used in all databases: (pneumoconiosis OR anthracosilicosis OR asbestosis OR berylliosis OR byssinosis OR siderosis OR silicosis OR silicotuberculosis) and (interleukin OR IL) and (polymorphism OR SNP OR genotype OR mutation OR variant). To fully investigate the association between IL gene variations and the risk of silicosis, we also manually screened the relative potential publications in the reference lists of included articles.

### 2.2 Selection and exclusion criteria

The following were the inclusion criteria: (1) studies with a case–control design to investigate the association between IL SNPs and pneumoconiosis susceptibility; (2) patients diagnosed clinically by chest X-ray and physical examinations based on the China National Diagnostic Criteria for Pneumoconiosis (GBZ 70–2002); and (3) the sufficient data on the genotypic frequencies of multiple IL genes.

The following were the exclusion criteria: (1) non-case–control studies; (2) reviews, case reports, meta-analyses, letters, and editorial articles; (3) duplicate publications; (4) cell and animal experiments; (5) lack of elaborate genotyping data; and (6) other gene type and additional IL gene polymorphisms.

### 2.3 Data extraction

Two researchers independently performed literature screening, data extraction, and literature quality assessment, and any disagreements between them was settled through a mutual discussion with a third analyst. Finally, the complete text of the included articles was reviewed, and the key results were extracted. The following data were collected from each study: first author, year of publication, country of the population, ethnicity, source of controls, genotyping methods, genotype distribution frequencies in cases and controls, and *p-*value for the Hardy–Weinberg equilibrium (HWE). Ethnicity was categorized as Caucasian and Asian, and the study designs were classified as population-based (PB) and hospital-based (HB) studies. We used the Newcastle–Ottawa scale (NOS) to evaluate the quality of the included articles. The score pattern comprised three aspects: queue selection (4 items, 0–4 stars), comparability of queues (1 item, 0–2 stars), and evaluation of results (3 items, 0–3 stars). A study with a score of at least 6 was considered as a high-quality literature. High NOS scores revealed a high-quality literature (51).

### 2.4 .Statistical analysis

Data analysis was conducted using Stata16.0 software (Stata Corp LP, TX, USA). Odds ratio (OR) and 95% confidence intervals (CIs) were used to detect the association between IL polymorphisms and silicosis. Then, the heterogeneity test was conducted. When *p* ≥ 0.05 or *I*^2^ < 50% was attained, it indicated that there was no statistical heterogeneity, and the fixed-effects model (FEM) was used to integrate the results. Otherwise, the random-effects model (REM) was used. Furthermore, a subgroup analysis was performed to determine more specific results based on ethnicity, disease types, sources of control, sample size of participants, and quality score. To evaluate the influence of each individual study on the overall results, a sensitivity analysis was performed by sequentially removing each study. Publication bias was assessed using the Begg’s rank correlation test and Egger’s linear regression test, and a *p*–value of <0.05 indicates an obvious publication bias.

### 2.5 False-positive report probability (FPRP) analysis

The probability of meaningful associations between IL gene SNPs and silicosis risk can be determined by conducting the FPRP analysis (52). In order to investigate the distinct correlations observed in this study, we adopted prior probabilities of 0.25, 0.1, 0.01, 0.001, and 0.0001 and computed the FPRP values as described previously. The association that reached an FPRP threshold of <0.2 was considered significant.

## 3 Results

### 3.1 Literature search and screening

**Figure 1:**
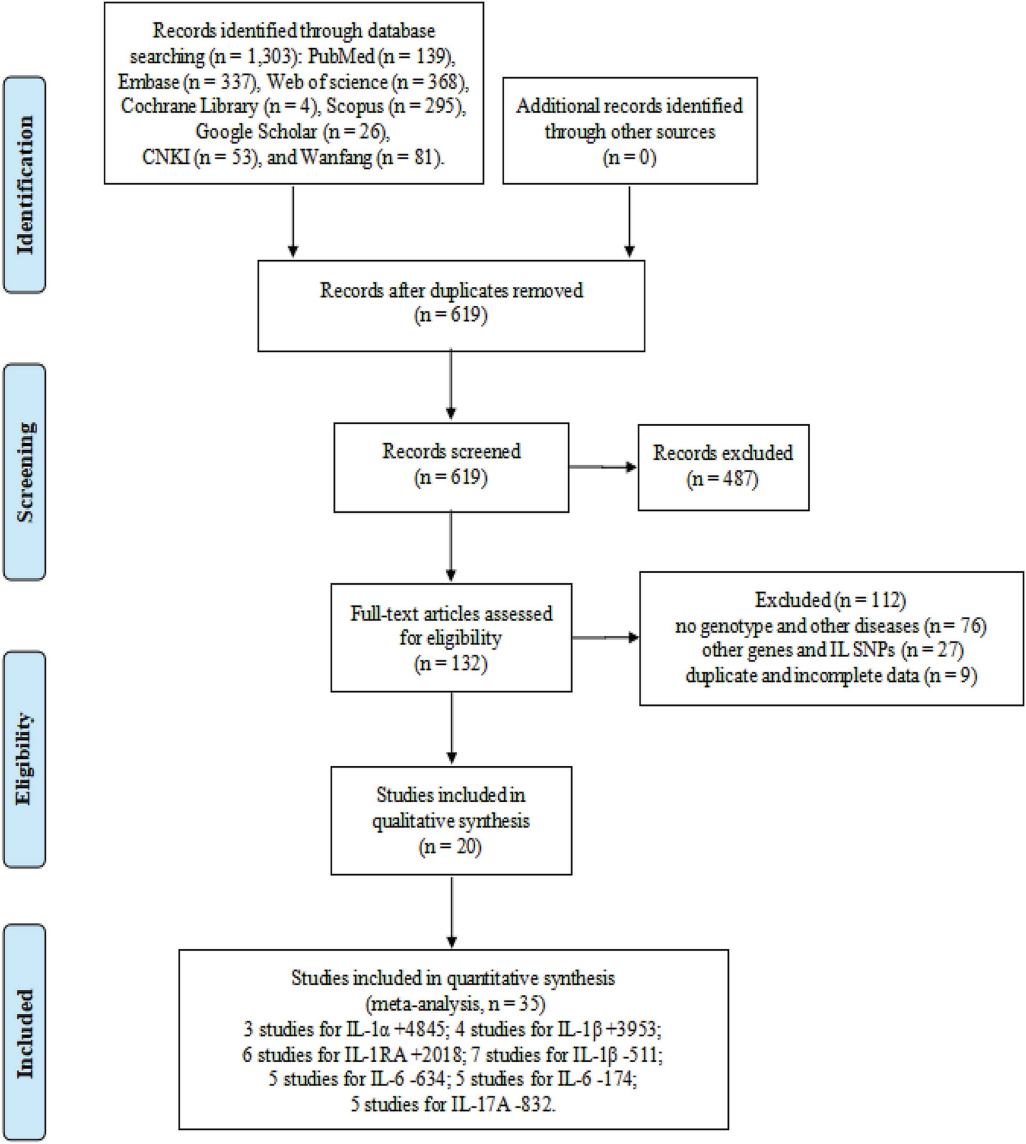
Flow diagram of the process of selecting eligible studies.

The flow diagram in Figure 1 shows the detailed literature search steps. The systematic search yielded 1,303 potential articles retrieved from the initial databases of PubMed (*n* = 139), Embase (*n* = 337), Web of Science (*n* = 368), Cochrane Library (*n* = 4), Scopus (*n* = 295), Google Scholar (*n* = 26), CNKI (*n* = 53), and Wanfang (*n* = 81). Moreover, no relevant studies were found in the above gray literature. After excluding 684 duplicates, 619 articles were considered for the meta-analysis. Then, we removed 487 articles after screening the titles and abstracts. Among these, 326 records were reviews, case reports, meta-analyses, letters, conference abstract, and editorial articles, and 161 records mainly focused on animal or *in vitro* studies. After carefully reviewing the full text, 112 studies were further excluded due to the following reasons: no genotype and other diseases (*n* = 76), duplicate and incomplete data (*n* = 9), and other genes and IL SNPs (*n* = 27). Finally, 20 eligible articles were retained for this meta-analysis (23, 31, 46–49, 53–66).

### 3.2 .Characteristics of the included studies

A total of 20 relevant articles including 35 studies were used for our study, which included 3 studies of IL-1α +4845G/T, 4 studies of IL-1β +3953C/T, 6 studies of IL-1RA +2018T/C, 7 studies of IL-1β -511C/T, 5 studies of IL-6 -634C/G, 5 studies of IL-6 -174G/C, and 5 studies of IL-17A -832A/G gene polymorphisms. Two disease types were mentioned in one article, so the article was evaluated as two independent case–control studies. Among these articles, 17 studies were from China, 2 studies from US, and 3 studies from other countries. Moreover, 4 studies were conducted on Caucasians and 18 studies were conducted on Asians. In the control group, 16 studies were population-based (PB) and 6 studies were hospital-based (HB). The characteristics of the included studies, such as genotyping method, sample size, type of disease, the Hardy–Weinberg equilibrium (HWE), and distribution of genotype frequency, are elaborated in Table 1 and Supplementary Table S1. The NOS scores of these studies ranged from 6 to 8, implying that all included studies were of high quality (Supplementary Table S2).

**Table 1:** Summary of characteristics of the studies included in our meta-analysis.

Author	Year	Country	Ethnicity	Sample size case/control	Genotyping methods	Source of control	NOS	Type of Disease
Yucesoy (46)	2001	USA	Caucasian	287/156	PCR-RFLP	PB	7	CWP
Zhai (64)	2001	China	Asian	99/160	PCR	HB	6	CWP
Fan XY_a (57)	2006	China	Asian	80/125	PCR-RFLP	PB	8	Silicosis
Fan XY_b (57)	2006	China	Asian	45/125	PCR-RFLP	PB	8	CWP
Liu_a (60)	2006	China	Asian	66/77	PCR-RFLP	PB	7	Silicosis
Liu_b (60)	2006	China	Asian	38/45	PCR-RFLP	PB	7	CWP
Wang DJ (61)	2006	China	Asian	75/137	PCR	PB	7	Silicosis
Ates (59)	2008	Turkey	Caucasian	67/92	PCR-RFLP	PB	7	CWP
Wu F (31)	2008	China	Asian	183/111	PCR-RFLP	PB	9	Silicosis
Yucesoy (63)	2008	USA	Caucasian	303/340	PCR-RFLP	PB	7	CWP
Fan XY (56)	2007	China	Asian	120/120	PCR-RFLP	PB	8	CWP
Dang ZC (55)	2012	China	Asian	120/120	PCR-RFLP	HB	6	CWP
Wang YW (23)	2012	China	Asian	68/68	PCR-RFLP	PB	8	Silicosis
Dang ZC (54)	2013	China	Asian	120/120	PCR-RFLP	HB	6	CWP
Chen Y_1 (48)	2014	China	Asian	113/116	PCR-RFLP	PB	7	Silicosis
Chen Y_2 (53)	2015	China	Asian	106/126	PCR-RFLP	PB	7	CWP
Han RH (58)	2015	China	Asian	693/689	PCR	PB	9	CWP
Hassani (49)	2017	Iran	Asian	48/62	PCR-RFLP	HB	7	Silicosis
Volobaev (47)	2018	Brazil	Caucasian	129/138	PCR	PB	8	CWP
Zhang Z (65)	2019	China	Asian	219/242	PCR-RFLP	PB	7	CWP
Zhou Y (66)	2022	China	Asian	45/45	PCR	HB	7	CWP
Xu XZ (62)	2022	China	Asian	160/150	PCR-RFLP	HB	7	CWP

PB, Population-based; HB, Hospital-based; PCR-RFLP, Polymerase chain reaction-restriction fragment length polymorphism; CWP, coal workers’ pneumoconiosis.

### 3.3 Meta-analysis of IL-1α +4845G/T, IL-1β +3953C/T, IL-1RA +2018T/C, and IL-1β -511C/T

**Figure 2:**
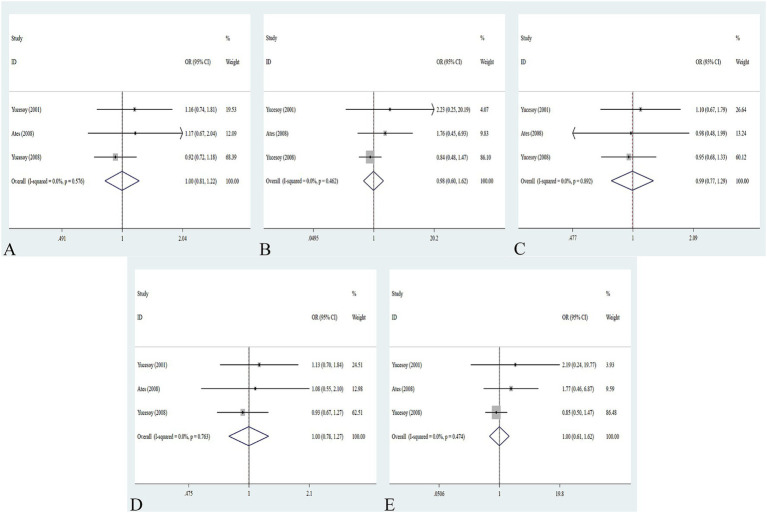
Association between IL-1α +4845G/T gene polymorphism and pneumoconiosis in all five models. **(A)** Allelic model; **(B)** dominant model; **(C)** heterozygous model; **(D)** homozygous model; and **(E)** recessive model.

**Figure 3:**
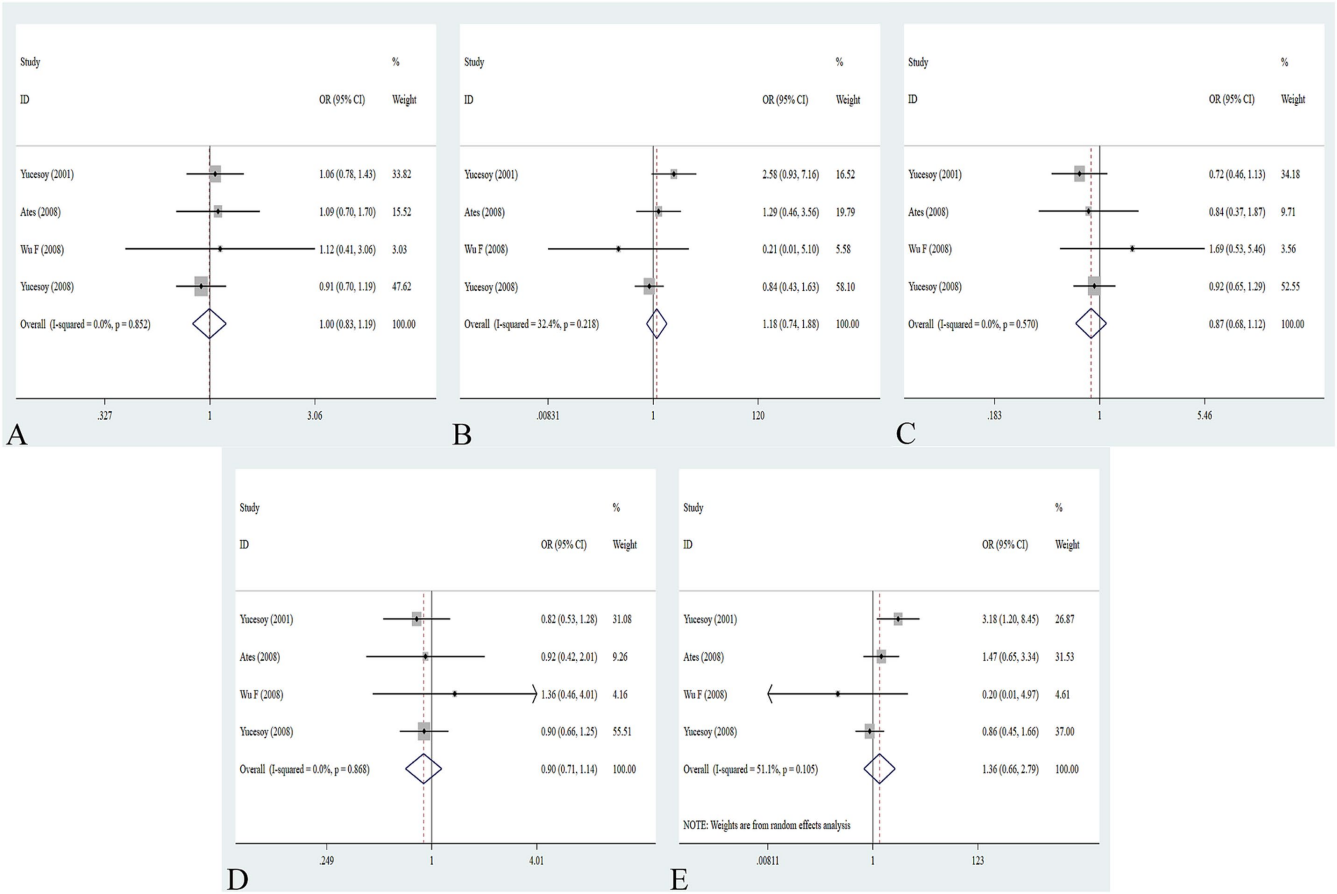
Association between IL-1β +3953C/T gene polymorphism and pneumoconiosis in all five models. **(A)** Allelic model; **(B)** dominant model; **(C)** heterozygous model; **(D)** homozygous model; and **(E)** recessive model.

**Figure 4:**
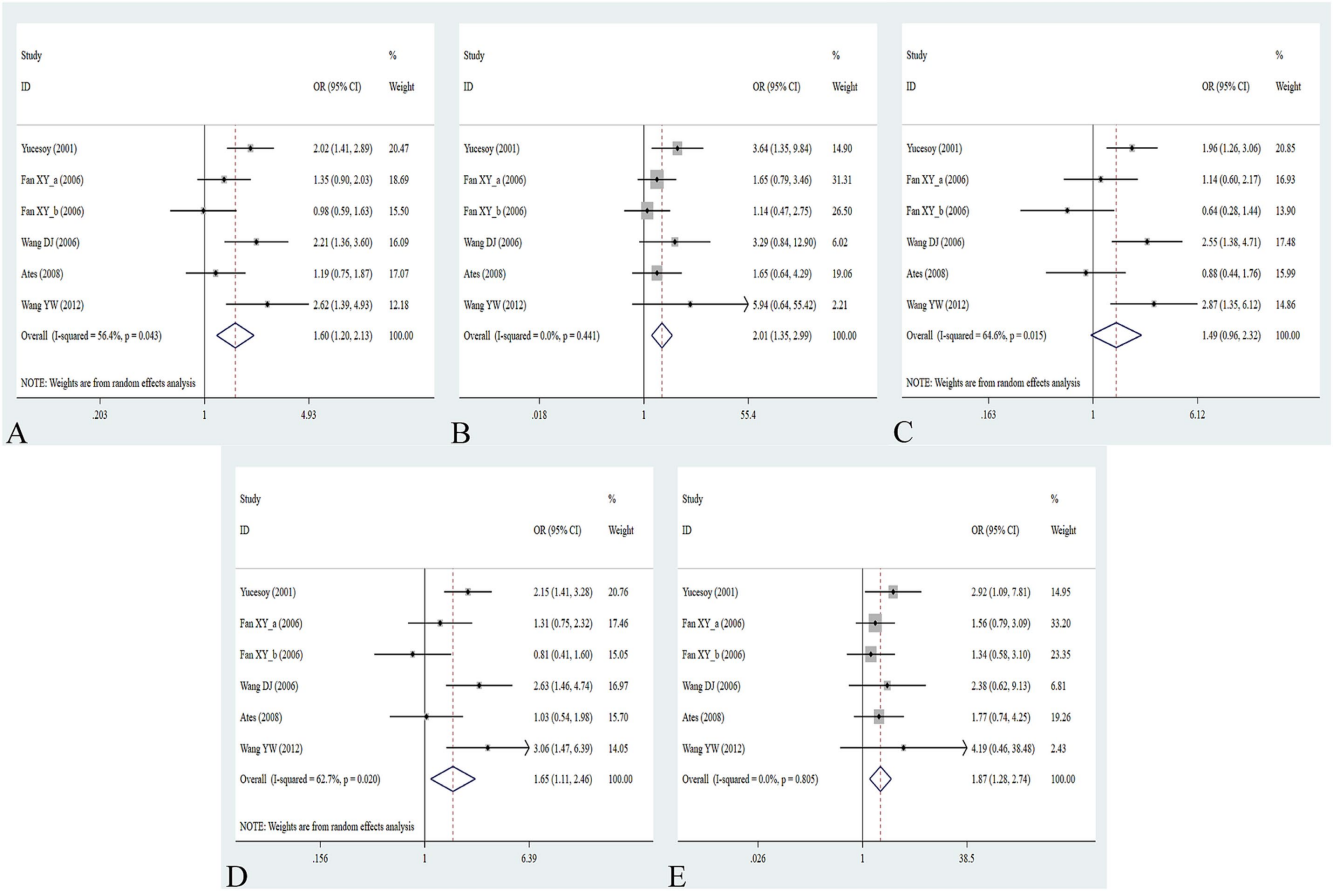
Association between IL-1RA +2018T/C gene polymorphism and pneumoconiosis in all five models. **(A)** Allelic model; **(B)** dominant model; **(C)** heterozygous model; **(D)** homozygous model; and **(E)** recessive model.

**Figure 5:**
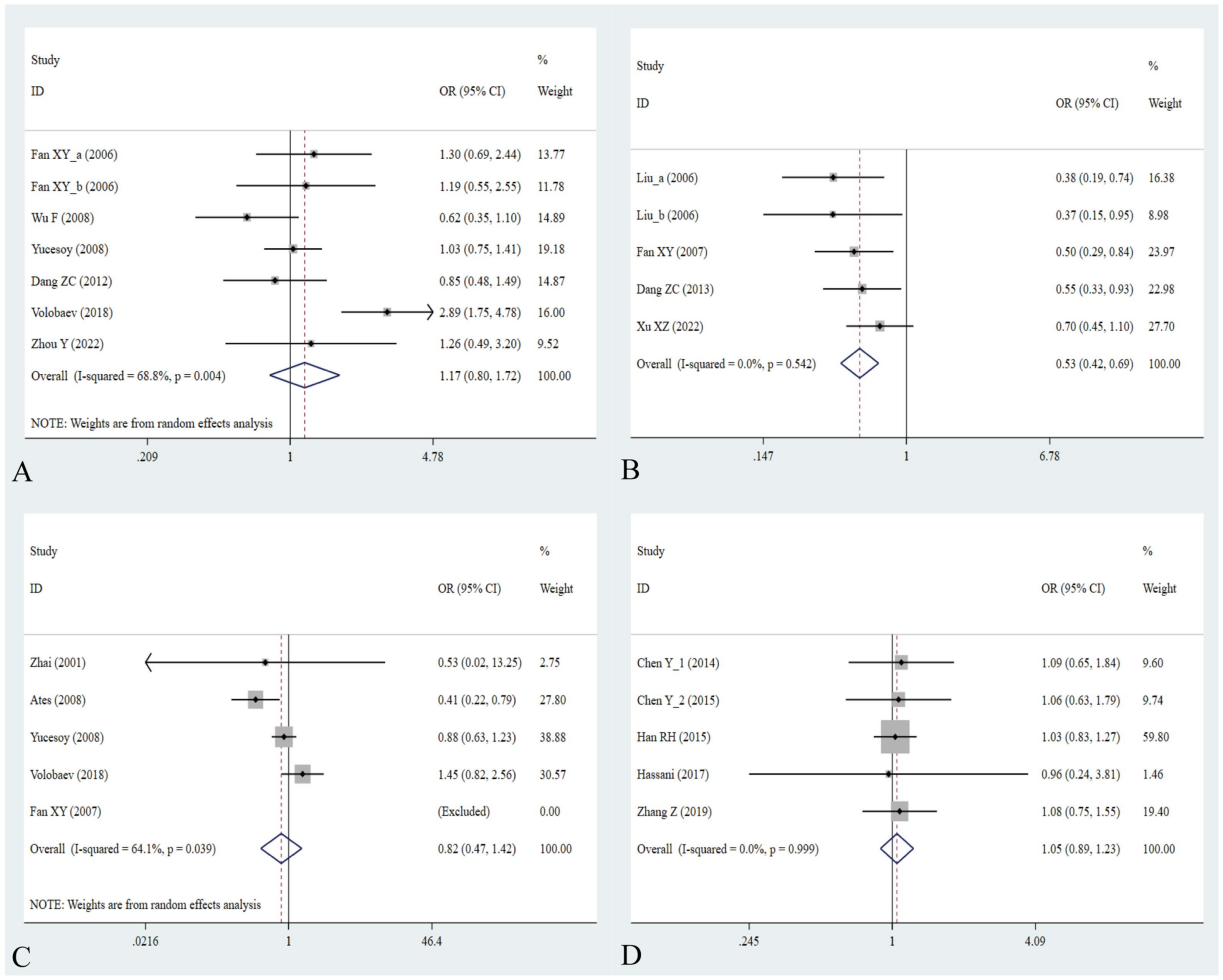
Association between five IL gene polymorphisms and pneumoconiosis in the dominant model. **(A)** IL-1β -511C/T polymorphism; **(B)** IL-6 -634C/G polymorphism; **(C)** IL-6 -174G/C polymorphism; and **(D)** IL-17A -832A/G polymorphism.

The association between the IL-1α +4845G/T, IL-1β +3953C/T, IL-1RA +2018T/C, and IL-1β -511C/T gene polymorphisms and pneumoconiosis was examined in 3 studies involving 645 patients and 581 controls, 4 studies involving 800 patients and 676 controls, 6 studies involving 609 patients and 704 controls, and 7 studies involving 905 patients and 1,006 controls, respectively. Overall, there were no significant associations of IL-1α +4845 (T vs. G: OR = 1.00, 95%CI = 0.81–1.22, *p* = 0.976; TT vs. GG: OR = 0.99, 95%CI = 0.60–1.62, *p* = 0.952; GT vs. GG: OR = 0.99, 95%CI = 0.77–1.29, *p* = 0.954; TT + GT vs. GG: OR = 1.00, 95%CI = 0.78–1.24, *p* = 0.979; TT vs. GT + GG: OR = 1.00, 95%CI = 0.61–1.62, *p* = 0.984, Figure 2) and IL-1β +3953 (T vs. C: OR = 1.00, 95%CI = 0.83–1.19, *p* = 0.957; TT vs. CC: OR = 1.18, 95%CI = 0.74–1.88, *p* = 0.485; CT vs. CC: OR = 0.87, 95%CI = 0.68–1.12, *p* = 0.277; TT + CT vs. CC: OR = 0.90, 95%CI = 0.71–1.14, *p* = 0.382; TT vs. CT + CC: OR = 1.36, 95%CI = 0.66–2.79, *p* = 0.408, Figure 3) with pneumoconiosis risk in all five genetic models. The IL-1RA +2018 polymorphism was evidently related to the pneumoconiosis risk (C vs. T: OR = 1.60, 95%CI = 1.20–2.13, *p* = 0.001; CC vs. TT: OR = 2.01, 95%CI = 1.35–2.99, *p* = 0.001; CC + CT vs. TT: OR = 1.65, 95%CI = 1.11–2.46, *p* = 0.013; CC vs. CT + TT: OR = 1.87, 95%CI = 1.28–2.74, *p* = 0.001, Figure 4). In addition, we found no obvious relation between the IL-1β-511 mutation and pneumoconiosis risk (T vs. C: OR = 1.22, 95%CI = 0.91–1.64, *p* = 0.176; TT vs. CC: OR = 1.46, 95%CI = 0.86–2.49, *p* = 0.160; CT vs. CC: OR = 1.06, 95%CI = 0.75–1.81, *p* = 0.737; TT + CT vs. CC: OR = 1.17, 95%CI = 0.80–1.72, *p* = 0.419; TT vs. CC + CT: OR = 1.42, 95%CI = 0.96–2.12, *p* = 0.083, Figure 5; Table 2).

**Table 2:** Meta-analysis results showing the association between IL gene SNPs and the risk of pneumoconiosis in overall analyses.

SNP	Model	OR (95% CI)	*P*	I^2^ (%)	*P_(H)_*	Effect model
IL-1α +4845G/T	Allelic Homozygous Heterozygous Dominant Recessive	1.00 (0.81, 1.22) 0.99 (0.60, 1.62) 0.99 (0.77, 1.29) 1.00 (0.78, 1.24) 1.00 (0.61, 1.62)	0.976 0.952 0.954 0.979 0.984	0.0 0.0 0.0 0.0 0.0	0.576 0.462 0.892 0.763 0.474	FEM FEM FEM FEM FEM
IL-1β +3953C/T	Allelic Homozygous Heterozygous Dominant Recessive	1.00 (0.83, 1.19) 1.18 (0.74, 1.88) 0.87 (0.68, 1.19) 0.90 (0.71, 1.14) 1.36 (0.66, 2.79)	0.957 0.485 0.277 0.382 0.408	0.0 32.4 0.0 0.0 51.1	0.852 0.218 0.570 0.868 0.105	FEM FEM FEM FEM REM
IL-1RA +2018T/C	Allelic Homozygous Heterozygous Dominant Recessive	1.60 (1.20, 2.13) 2.01 (1.35, 2.99) 1.49 (0.96, 2.32) 1.65 (1.11, 2.46) 1.87 (1.28, 2.74)	0.001^*^0.001^*^ 0.074 0.013^*^0.001^*^	56.4 0.0 64.6 62.7 0.0	0.043 0.441 0.015 0.020 0.295	REM FEM REM REM FME
IL-1β -511C/T	Allelic Homozygous Heterozygous Dominant Recessive	1.22 (0.91, 1.64) 1.46 (0.86, 2.49) 1.06 (0.75, 1.81) 1.17 (0.80, 1.72) 1.42 (0.96, 2.12)	0.176 0.160 0.737 0.419 0.083	77.0 72.6 52.7 68.8 64.1	0.000 0.004 0.048 0.004 0.010	REM REM REM REM RME
IL-6 -634C/G	Allelic Homozygous Heterozygous Dominant Recessive	0.62 (0.51, 0.76) 0.57 (0.40, 0.83) 0.50 (0.37, 0.67 ) 0.54 (0.42, 0.69) 0.71 (0.50, 1.01)	0.000^*^ 0.003^*^ 0.000^*^ 0.000^*^ 0.056	0.0 0.0 39.5 0.0 0.0	0.837 0.721 0.158 0.542 0.494	FEM FEM FEM FEM FEM
IL-6 -174G/C	Allelic Homozygous Heterozygous Dominant Recessive	0.95 (0.79, 1.13) 1.00 (0.70, 1.44) 0.75 (0.37, 1.50) 0.82 (0.47, 1.42) 1.05 (0.76, 1.45)	0.538 0.983 0.413 0.477 0.768	11.6 0.0 73.8 64.1 24.7	0.335 0.405 0.009 0.039 0.265	FME FME REM REM FME
IL-17A -832A/G	Allelic Homozygous Heterozygous Dominant Recessive	1.02 (0.90, 1.15) 0.94 (0.69, 1.29) 1.07 (0.90, 1.26) 1.05 (0.89, 1.23) 0.96 (0.72, 1.28)	0.761 0.702 0.455 0.579 0.780	0.0 0.0 0.0 0.0 0.0	0.977 0.990 0.997 0.999 0.862	FEM FEM FEM FEM FME

When conducting the subgroup analyses based on ethnicity, type of disease, source of control, NOS score, and sample size, we did not find any association between IL-1α +4845, IL-1β +3953, and IL-1β -511 gene polymorphisms and pneumoconiosis. As for IL-1RA +2018, the C-allele variant significantly increased the pneumoconiosis risk among Asians (C vs. T: OR = 1.62, 95%CI = 1.07–2.45, *p* = 0.022; CC vs. TT: OR = 1.74, 95%CI = 1.06–2.85, *p* = 0.030; CC vs. CT + TT: OR = 1.66, 95%CI = 1.03–2.68, *p* = 0.036) and Caucasians (CC vs. TT: OR = 2.53, 95%CI = 1.29–4.95, *p* = 0.007; CC vs. CT + TT: OR = 2.27, 95%CI = 1.19–4.34, *p* = 0.013). An obvious correlation was found between the IL-1RA +2018 variant and increased predisposition to silicosis (C vs. T: OR = 1.89, 95%CI = 1.26–2.84, *p* = 0.002; CC vs. TT: OR = 2.13, 95%CI = 1.16–3.97, *p* = 0.015; CC + CT vs. TT: OR = 2.13, 95%CI = 1.26–3.60, *p* = 0.005; CC vs. CT + TT: OR = 1.81, 95%CI = 1.03–3.29, *p* = 0.040) and CWP (CC vs. TT: OR = 1.92, 95%CI = 1.14–3.23, *p* = 0.015; CC vs. CT + TT: OR = 1.89, 95%CI = 1.14–3.13, *p* = 0.013). Meanwhile, evident correlations between IL-1RA +2018 and pneumoconiosis were examined in subgroups of PB, lower quality scores, and large sample sizes. The results of the heterogeneity test revealed that heterogeneity existed in IL-1RA +2018 and IL-1β -511. With regard to IL-1RA +2018, heterogeneity mainly appeared in the allelic, heterozygous, and dominant models. As for IL-1β -511, heterogeneity significantly diminished or disappeared in the subgroups of HB and lower quality scores.

### 3.4 Meta-analysis of IL-6 -634C/G, IL-6 -174G/C, and IL-17A -832A/G

The association between the IL-6 -634C/G, IL-6 -174G/C, and IL-17A -832A/G gene polymorphisms and pneumoconiosis was examined in 5 studies involving 504 patients and 512 controls, 5 studies involving 660 patients and 848 controls, and 5 studies involving 1,179 patients and 1,235 controls. The IL-6 -634 variant was significantly associated with an enhanced pneumoconiosis risk in the allelic, homozygous, heterozygous, and dominant models (G vs. C: OR = 0.62, 95%CI = 0.51–0.76, *p* = 0.000; GG vs. CC: OR = 0.57, 95%CI = 0.40–0.83, *p* = 0.003; GC vs. CC: OR = 0.50, 95%CI = 0.37–0.67, *p* = 0.000; GG + GC vs. CC: OR = 0.54, 95%CI = 0.42–0.69, *p* = 0.000). The results of overall analyses disclosed that IL-6 -174 was not related to the pneumoconiosis susceptibility (C vs. G: OR = 0.95, 95%CI = 0.79-1.13, *p* = 0.538; CC vs. GG: OR = 1.00, 95%CI = 0.70-1.44, *p* = 0.983; CG vs. GG: OR = 0.75, 95%CI = 0.37-1.50, *p* = 0.413; CC+CG vs. GG: OR = 0.82, 95%CI = 0.47-1.42, *p* = 0.477; CC vs. CG+GG: OR = 1.05, 95%CI = 0.76-1.45, *p* = 0.768). As for the IL-17A -832 variant, we did not find any remarkable relevance in the five genetic models (G vs. A: OR = 1.02, 95%CI = 0.90–1.1, *p* = 0.761; GG vs. AA: OR = 0.94, 95%CI = 0.69–1.29, *p* = 0.702; GA vs. AA: OR = 1.07, 95%CI = 0.90–1.26, *p* = 0.455; GG + GA vs. AA: OR = 1.05, 95%CI = 0.89–1.23, *p* = 0.579; GG vs. GA + AA: OR = 0.96, 95%CI = 0.72–1.28, *p* = 0.780, Figure 5; Table 2). The IL-6 -634G-allele remarkably decreased the pneumoconiosis susceptibility among Asians (G vs. C: OR = 0.62, 95%CI = 0.51–0.76, *p* = 0.000; GG vs. CC: OR = 0.57, 95%CI = 0.40–0.83, *p* = 0.003; GC vs. CC: OR = 0.50, 95%CI = 0.37–0.67, *p* = 0.000; GG + GC vs. CC: OR = 0.54, 95%CI = 0.42–0.69, *p* = 0.000, Table 3), indicating that the G-allele might be a protective factor in the Asian population. Moreover, we found remarkable association between the IL-6 -634 mutant and pneumoconiosis based on the subgroups of PB, HB, and low and high scores. Further subgroup analyses manifested no significant association between the IL-6 -174 and IL-17A -832 gene polymorphisms and pneumoconiosis. The results of heterogeneity test exhibited *I*^2^ vales of 73.8 and 64.1, suggesting that heterogeneity clearly existed in the heterozygous and dominant models of the IL-6 -174G/C polymorphism. Therefore, a random-effects model was employed to investigate the association. Notably, the analysis revealed no heterogeneity in the IL-6 -634 and IL-17A -832A/G gene polymorphisms, leading to the use of a fixed-effects model.

**Table 3:** Meta-analysis results showing the association between IL gene polymorphisms and the risk of pneumoconiosis based on subgroup analyses.

Locus	No.	Allele		Homozygote		Heterozygote		Dominant		Recessive	
		OR (95%CI) *P*	I^2^ (%)	OR (95%CI) *P*	I^2^ (%)	OR (95%CI) *P*	I^2^ (%)	OR (95%CI) *P*	I^2^ (%)	OR (95%CI) *P*	I^2^ (%)
IL-1α + 4845G/T gene polymorphism											
Ethnicity											
Caucasian	3	1.00 (0.81, 1.22) 0.976	0.0	0.99 (0.60, 1.62) 0.952	0.0	0.99 (0.77, 1.29) 0.954	0.0	1.00 (0.78, 1.24) 0.979	0.0	1.00 (0.61, 1.62) 0.984	0.0
Type of disease											
CWP	3	1.00 (0.81, 1.22) 0.976	0.0	0.99 (0.60, 1.62) 0.952	0.0	0.99 (0.77, 1.29) 0.954	0.0	1.00 (0.78, 1.24) 0.979	0.0	1.00 (0.61, 1.62) 0.984	0.0
Source of control											
PB	3	1.00 (0.81, 1.22) 0.976	0.0	0.99 (0.60, 1.62) 0.952	0.0	0.99 (0.77, 1.29) 0.954	0.0	1.00 (0.78, 1.24) 0.979	0.0	1.00 (0.61, 1.62) 0.984	0.0
NOS scores											
N2	3	1.00 (0.81, 1.22) 0.976	0.0	0.99 (0.60, 1.62) 0.952	0.0	0.99 (0.77, 1.29) 0.954	0.0	1.00 (0.78, 1.24) 0.979	0.0	1.00 (0.61, 1.62) 0.984	0.0
Sample size											
S1	1	1.17 (0.67, 2.04) 0.591	_	1.76 (0.45, 6.93) 0.418	_	0.98 (0.48, 1.99) 0.946	_	1.08 (0.56, 2.10) 0.820	_	1.77 (0.46, 6.87) 0.732	_
S2	2	0.97 (0.78, 1.21) 0.811	0.0	0.90 (0.53, 1.54) 0.701	0.0	0.96 (0.75, 1.32) 0.972	0.0	0.98 (0.76, 1.28) 0.906	0.0	0.91 (0.54, 1.54) 0.407	0.0
IL-1β + 3953C/T gene polymorphism											
Ethnicity											
Caucasian	3	0.99 (0.83, 1.19) 0.925	0.0	1.24 (0.77, 1.98) 0.377	39.6	0.84 (0.65, 1.09) 0.184	0.0	0.88 (0.69, 1.13) 0.304	0.0	1.48 (0.72, 3.06) 0.287	58.6
Asian	1	1.12 (1.41, 3.06) 0.832	_	0.21 (0.01, 5.10) 0.334	_	1.70 (0.53, 5.46) 0.377	_	1.36 (0.46, 4.01) 0.582	_	0.20 (0.01, 4.97) 0.327	_
Type of disease											
CWP	3	0.99 (0.83, 1.19) 0.925	0.0	1.24 (0.77, 1.98) 0.377	39.6	0.84 (0.65, 1.09) 0.184	0.0	0.88 (0.69, 1.13) 0.304	0.0	1.48 (0.72, 3.06) 0.287	58.6
Silicosis	1	1.12 (1.41, 3.06) 0.832	_	0.21 (0.01, 5.10) 0.334	_	1.70 (0.53, 5.46) 0.377	_	1.36 (0.46, 4.01) 0.582	_	0.20 (0.01, 4.97) 0.327	_
Source of control											
PB	3	0.96 (0.77, 1.20) 0.738	0.0	1.18 (0.74, 1.88) 0.485	32.4	0.87 (0.68, 1.1+) 0.277	0.0	0.90 (0.71, 1.14) 0.382	0.0	1.36 (0.66, 2.79) 0.408	51.1
NOS scores											
N1	3	0.99 (0.83, 1.19) 0.925	0.0	1.24 (0.77, 1.98) 0.377	39.6	0.84 (0.65, 1.09) 0.184	0.0	0.88 (0.69, 1.13) 0.304	0.0	1.48 (0.72, 3.06) 0.287	58.6
N2	1	1.12 (1.41, 3.06) 0.832	_	0.21 (0.01, 5.10) 0.334	_	1.70 (0.53, 5.46) 0.377	_	1.36 (0.46, 4.01) 0.582	_	0.20 (0.01, 4.97) 0.327	_
Sample size											
S2	2	1.10 (0.73, 1.65) 0.783	0.0	1.22 (0.72, 2.09) 0.457	69.7	0.84 (0.64, 1.10) 0.209	0.0	0.87 (0.67, 1.13) 0.311	0.0	1.57 (0.44, 5.67) 0.489	79.1
S1	2	0.97 (0.80, 1.19) 0.663	0.0	1.05 (0.41, 2.70) 0.922	12.5	1.07 (0.56, 2.05) 0.848	0.0	1.06 (0.56, 1.98) 0.866	0.0	1.02 (0.22, 4.66) 0.983	28.4
IL-1RA + 2018T/C gene polymorphism											
Ethnicity											
Caucasian	2	1.58 (0.94, 2.65) 0.085	69.0	2.53 (1.29, 4.95) 0.007^*^	21.7	1.38 (0.63, 3.00) 0.419	72.5	1.56 (0.76, 3.19) 0.226	71.2	2.27 (1.19, 4.34) 0.013^*^	0.0
Asian	4	1.62 (1.07, 2.45) 0.022^*^	63.3	1.74 (1.06, 2.87) 0.030^*^	0.0	1.55 (0.81, 2.98) 0.190	71.3	1.70 (0.95, 3.05) 0.073	69.8	1.66 (1.03, 2.68) 0.036^*^	0.0
Type of disease											
CWP	3	1.37 (0.88, 2.14) 0.169	68.1	1.92 (1.14, 3.23) 0.015^*^	34.0	1.10 (0.54, 2.24) 0.793	73.1	1.27 (0.67, 2.41) 0.458	72.4	1.89 (1.14, 3.13) 0.013^*^	0.0
Silicosis	3	1.89 (1.26, 2.84) 0.002^*^	49.2	2.13 (1.16, 3.97) 0.015^*^	0.0	2.00 (1.13, 3.55) 0.074	54.7	2.13 (1.26, 3.60) 0.005^*^	52.3	1.81 (1.03. 3.29) 0.040^*^	0.0
Source of control											
PB	6	1.60 (1.20, 2.13) 0.001^*^	56.4	2.53 (1.29, 4.95) 0.007^*^	21.7	1.49 (0.96, 2.32) 0.074	64.6	1.49 (0.96, 2.32) 0.074	64.6	1.87 (1.28, 2.74) 0.001^*^	0.0
NOS scores											
N1	3	1.75 (1.22, 2.52) 0.002^*^	53.0	2.64 (1.44, 4.84) 0.002^*^	0.0	1.70 (0.98, 2.94) 0.060	63.4	1.86 (1.13, 3.05) 0.014^*^	59.1	2.29 (1.28, 4.10) 0.005^*^	0.0
N2	3	1.46 (0.89, 2.39) 0.132	64.5	1.58 (0.92, 2.71) 0.095	_	1.29 (0.57, 2.90) 0.540	72.6	1.46 (0.72, 2.96) 0.294	71.0	1.58 (0.95, 2.63) 0.078	0.0
Sample size											
S2	1	2.02 (1.41, 2.89) 0.000^*^	_	3.64 (1.35, 9.84) 0.011^*^	_	1.96 (1.26, 3.06) 0.003^*^	_	2.16 (1.41, 3.29) 0.000^*^	_	2.92 (1.09, 7.81) 0.033^*^	_
S1	5	1.51 (1.08, 2.11) 0.015^*^	56.9	1.72 (1.10, 2.67) 0.016^*^	0.0	1.39 (0.79, 2.42) 0.253	68.7	1.54 (0.94, 2.52) 0.084	65.9	1.69 (1.11, 2.56) 0.014^*^	0.0
IL-1β -511C/T gene polymorphism											
Ethnicity											
Asian	5	1.07 (0.81, 1.41) 0.623	53.3	1.12 (0.69, 1.83) 0.646	49.9	0.85 (0.61, 1.19) 0.342	0.0	0.93 (0.70, 1.25) 0.647	0.0	1.22 (0.81, 1.84) 0.351	52.5
Caucasian	2	1.60 (0.72, 3.56) 0.248	92.6	2.90 (0.50, 16.73) 0.234	90.4	1.49 (0.62, 3.58) 0.375	87.5	1.69 (0.62, 4.65) 0.307	91.4	2.30 (0.63, 8.43) 0.207	84.2
Type of disease											
Silicosis	2	0.99 (0.58, 1.71) 0.984	77.1	0.98 (0.33, 2.93) 0.976	77.4	0.83 (0.49, 1.41) 0.492	27.6	0.89 (0.43, 1.81) 0.740	65.0	1.09 (0.52, 2.28) 0.812	67.0
CWP	5	1.34 (0.93, 1.93) 0.120	78.5	1.74 (0.90, 3.34) 0.098	73.9	1.17 (0.75, 1.81) 0.495	58.4	1.31 (0.82, 2.10) 0.261	71.4	1.62 (0.97. 2.70) 0.067	67.6
Source of control											
PB	5	1.31 (0.90, 1.90) 0.159	82.8	1.72 (0.84, 3.53) 0.142	79.3	1.09 (0.70, 1.69) 0.714	67.7	1.24 (0.75, 2.04) 0.402	77.6	1.65 (0.97, 2.80) 0.064	79.0
HB	2	0.97 (0.70, 1.35) 0.853	8.0	0.94 (0.56, 1.58) 0.827	0.0	0.93 (0.51, 1.71) 0.820	0.0	0.94 (0.58, 1.53) 0.804	0.0	0.98 (0.63, 1.50) 0.912	0.0
NOS scores											
N2	6	1.39 (0.82, 2.34) 0.218	85.9	1.93 (0.69, 5.41) 0.212	84.1	1.12 (0.59, 2.11) 0.748	74.0	1.30 (0.65, 2.63) 0.460	81.4	1.84 (0.88, 3.88) 0.107	77.9
N1	1	1.04 (0.86, 1.25) 0.691	0.0	1.10 (0.77, 1.56) 0.607	0.0	0.96 (0.72, 1.29) 0.801	0.0	1.00 (0.77, 1.31) 0.982	0.0	1.11 (0.81, 1.51) 0.518	0.0
Sample size											
S1	6	1.26 (0.86, 1.84) 0.231	80.3	1.54 (0.78, 3.05) 0.215	77.1	1.08 (0.68, 1.69) 0.751	58.9	1.20 (0.73, 1.98) 0.467	72.8	1.49 (0.90, 2.46) 0.121	70.1
S2	1	1.08 (0.86, 1.35) 0.506	_	1.25 (0.77, 2.02) 0.365	_	0.97 (0.70, 1.36) 0.870	_	1.03 (0.75, 1.42) 0.851	_	1.27 (0.81, 1.98) 0.297	_
IL-6 -634C/G gene polymorphism											
Ethnicity											
Asian	5	0.62 (0.51, 0.76) 0.000^*^	0.0	0.57 (0.40, 0.83) 0.003^*^	0.0	0.50 (0.37, 0.67) 0.000^*^	39.5	0.54 (0.42, 0.69) 0.000^*^	0.0	0.71 (0.50, 1.01) 0.056	0.0
Type of disease											
Silicosis	1	0.55 (0.33, 0.93) 0.025^*^	_	0.61 (0.23, 1.65) 0.334	_	0.30 (0.14, 0.65) 0.002^*^	_	0.38 (0.19, 0.74) 0.005^*^	_	0.95 (0.37, 2.45) 0.911	_
CWP	4	0.63 (0.51, 0.78) 0.000^*^	0.0	0.64 (0.41, 0.98) 0.042^*^	0.0	0.55 (0.40, 0.76) 0.000^*^	34.2	0.57 (0.43, 0.74) 0.000^*^	0.0	0.68 (0.46, 0.49) 0.044^*^	0.0
Source of control											
PB	3	0.59 (0.44, 0.79) 0.000^*^	0.0	0.67 (0.38, 1.17) 0.158	0.0	0.35 (0.23, 0.54) 0.000^*^	0.0	0.43 (0.30, 0.63) 0.000^*^	0.0	0.94 (0.54, 1.61) 0.813	0.0
HB	2	0.65 (0.50, 0.84) 0.010^*^	0.0	0.52 (0.32, 0.83) 0.007^*^	0.0	0.69 (0.46, 1.05) 0.081	30.9	0.63 (0.45, 0.89) 0.008^*^	0.0	0.58 (0.36, 0.92) 0.021^*^	7.1
NOS scores											
N1	4	0.61 (0.49, 0.76) 0.000^*^	0.0	0.52 (0.34, 0.79) 0.002^*^	0.0	0.54 (0.38, 0.76) 0.000^*^	47.3	0.55 (0.41, 0.73) 0.000^*^	0.0	0.63 (0.42, 0.94) 0.024^*^	0.0
N2	1	0.66 (0.44, 1.00) 0.050	_	0.81 (0.37, 1.76) 0.588	_	0.39 (0.21, 1.71) 0.002^*^	_	0.50 (0.29, 0.84) 0.009^*^	_	1.08 (0.51, 2.29) 0.847	_
Sample size											
S1	5	0.62 (0.51, 0.76) 0.000^*^	0.0	0.57 (0.40, 0.83) 0.003^*^	0.0	0.50 (0.37, 0.67) 0.000^*^	39.5	0.54 (0.42, 0.69) 0.000^*^	0.0	0.71 (0.50, 1.01) 0.056	0.0
IL-6 -174G/C gene polymorphism											
Ethnicity											
Asian	1	0.59 (0.02, 14.56) 0.747	_	_	_	0.59 (0.02, 14.57) 0.745	_	0.59 (0.02, 14.57) 0.745	_	_	_
Caucasian	3	0.95 (0.79, 1.13) 0.553	38.9	1.00 (0.70, 1.44) 0.983	0.0	0.76 (0.36, 1.60) 0.464	82.4	0.83 (0.46, 1.50) 0.531	75.8	1.05 (0.76, 1.45) 0.768	24.7
Type of disease											
CWP	4	0.95 (0.79, 1.13) 0.541	10.6	1.00 (0.70, 1.44) 0.983	0.0	0.84 (0.64, 1.10) 0.201	73.8	0.82 (0.48, 1.42) 0.482	63.9	1.05 (0.76, 1.45) 0.768	24.7
Source of control											
HB	1	0.59 (0.02, 14.56) 0.747	_	_	_	0.59 (0.02, 14.57) 0.745	_	0.59 (0.02, 14.57) 0.745	_	_	_
PB	3	0.95 (0.79, 1.13) 0.553	38.9	1.00 (0.70, 1.44) 0.983	0.0	0.76 (0.36, 1.60) 0.464	82.4	0.83 (0.46, 1.50) 0.531	75.8	1.05 (0.76, 1.45) 0.768	24.7
NOS scores											
N1	3	0.86 (0.70, 1.07) 0.169	0.0	0.88 (0.58, 1.34) 0.547	0.0	0.55 (0.22, 1.42) 0.216	73.0	0.65 (0.35, 1.20) 0.165	52.6	1.01 (0.68, 1.49) 0.971	60.1
N2	1	1.22 (0.86, 1.73) 0.268	_	1.47 (0.72, 3.00) 0.290	_	1.44 (0.79, 2.62) 0.230	_	1.45 (0.82, 2.56) 0.198	_	1.16 (0.64, 2.10) 0.627	_
Sample size											
S1	3	1.01 (0.77, 1.34) 0.931	32.1	1.36 (0.76, 2.44) 0.307	0.0	0.66 (0.17, 2.49) 0.537	81.9	0.76 (0.26, 2.24) 0.662	75.9	1.36 (0.82, 2.24) 0.335	24.3
S2	1	0.90 (0.71, 1.14) 0.382	_	0.83 (0.52, 1.32) 0.434	_	0.90 (0.63, 1.29) 0.5754	_	0.88 (0.63, 1.23) 0.452	_	0.88 (0.57, 1.34) 0.541	_
IL-17A -832A/G gene polymorphism											
Ethnicity											
Asian	5	1.02 (0.90, 1.15) 0.761	0.0	0.94 (0.69, 1.28) 0.702	0.0	1.07 (0.90, 1.28) 0.455	0.0	1.05 (0.89, 1.23) 0.579	0.0	0.96 (0.72, 1.23) 0.780	0.0
Type of disease											
Silicosis	2	1.10 (0.80, 1.53) 0.554	0.0	1.11 (0.49, 2.53) 0.799	0.0	1.06 (0.64, 1.75) 0.828	0.0	1.08 (0.66, 1.75) 0.765	0.0	1.25 (0.68, 2.28) 0.470	0.0
CWP	3	1.01 (0.88, 1.15) 0.932	0.0	0.91 (0.65, 1.28) 0.603	0.0	1.07 (0.89, 1.28) 0.455	0.0	1.04 (0.88, 1.24) 0.629	0.0	0.89 (0.64, 1.23) 0.475	0.0
Source of control											
PB	4	1.01 (0.89, 1.15) 0.864	0.0	0.93 (0.67, 1.28) 0.656	0.0	1.07 (0.90, 1.27) 0.433	0.0	1.05 (0.89, 1.24) 0.572	0.0	0.90 (0.66, 1.23) 0.511	0.0
HB	1	1.19 (0.68, 2.10) 0.536	_	1.19 (0.28, 5.06) 0.811	_	0.82 (0.20, 3.39) 0.785	_	0.97 (0.25, 3.81) 0.959	_	1.41 (0.65, 3.06) 0.379	_
NOS scores											
N1	4	1.06 (0.88, 1.28) 0.569	0.0	1.03 (0.63, 1.69) 0.909	0.0	1.08 (0.83, 1.40) 0.580	0.0	1.07 (0.83, 1.38) 0.587	0.0	1.09 (0.71, 1.66) 0.704	0.0
N2	1	0.99 (0.84, 1.15) 0.924	_	0.89 (0.59, 1.33) 0.556	_	1.06 (0.85, 1.32) 0.610	_	1.03 (0.83, 1.27) 0.786	_	0.86 (0.58, 1.28) 0.462	_
Sample size											
S1	3	1.07 (0.83, 1.38) 0.602	0.0	1.04 (0.54, 2.02) 0.903	0.0	1.07 (0.74, 1.54) 0.719	0.0	1.07 (0.75, 1.53) 0.705	0.0	1.15 (0.68, 1.95) 0.599	0.0
S2	2	1.00 (0.87, 1.16) 0.957	0.0	0.91 (0.64, 1.31) 0.617	0.0	1.07 (0.88, 1.29) 0.513	0.0	1.04 (0.87, 1.25) 0.668	0.0	0.89 (0.63, 1.25) 0.496	0.0

^*^p < 0.05.

### 3.5 Sensitivity analysis and publication bias

**Figure 6:**
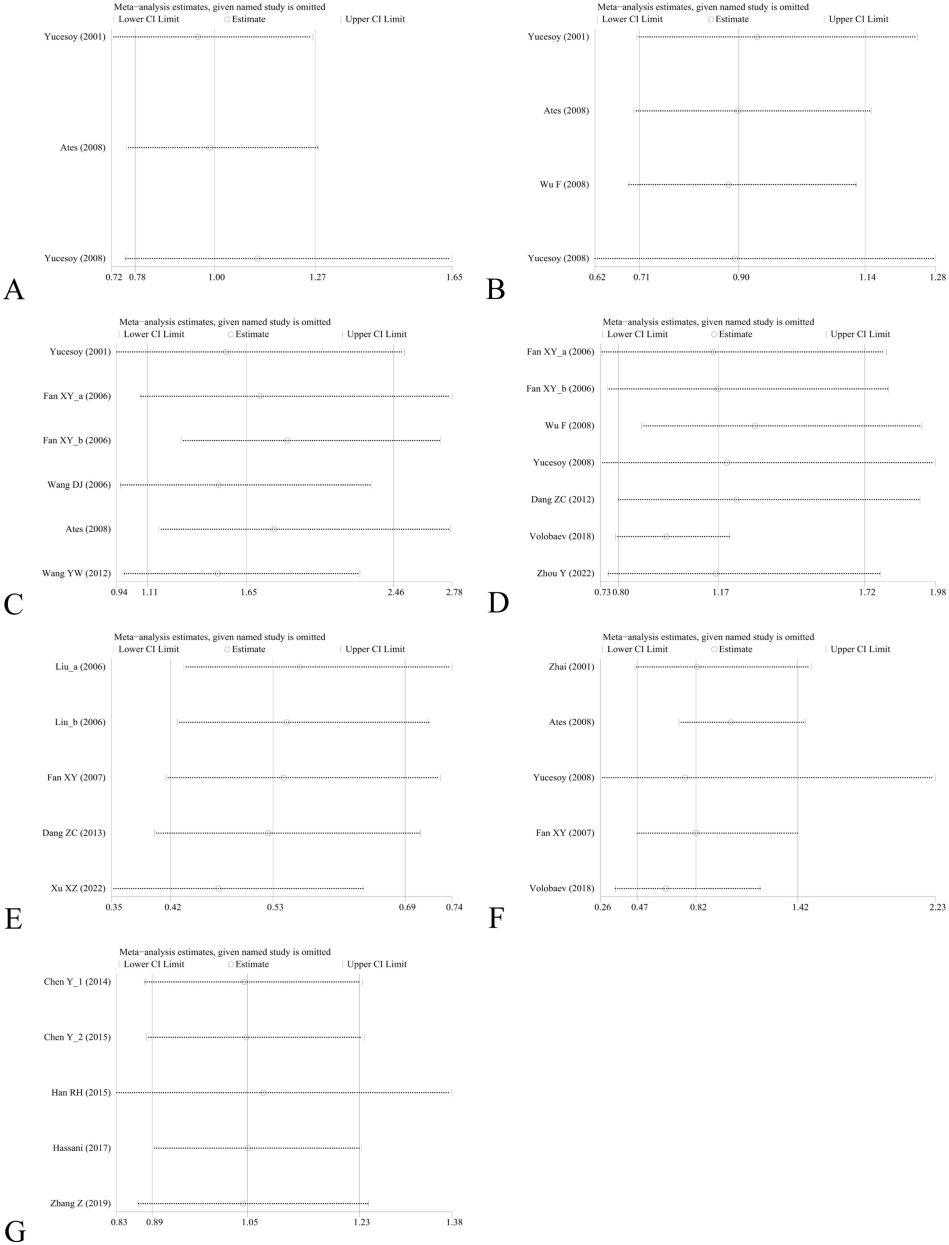
Sensitivity analysis by removing one study at a time to assess the influence of individual datasets on the pooled ORs for various IL gene polymorphisms using the dominant model. **(A)** IL-1α +4845G/T; **(B)** IL-1β +3953C/T; **(C)** IL-1RA +2018T/C; **(D)** IL-1β -511C/T; **(E)** IL-6 -634C/G; **(F)** IL-6 -174G/C; and **(G)** IL-17A -832A/G.

**Figure 7:**
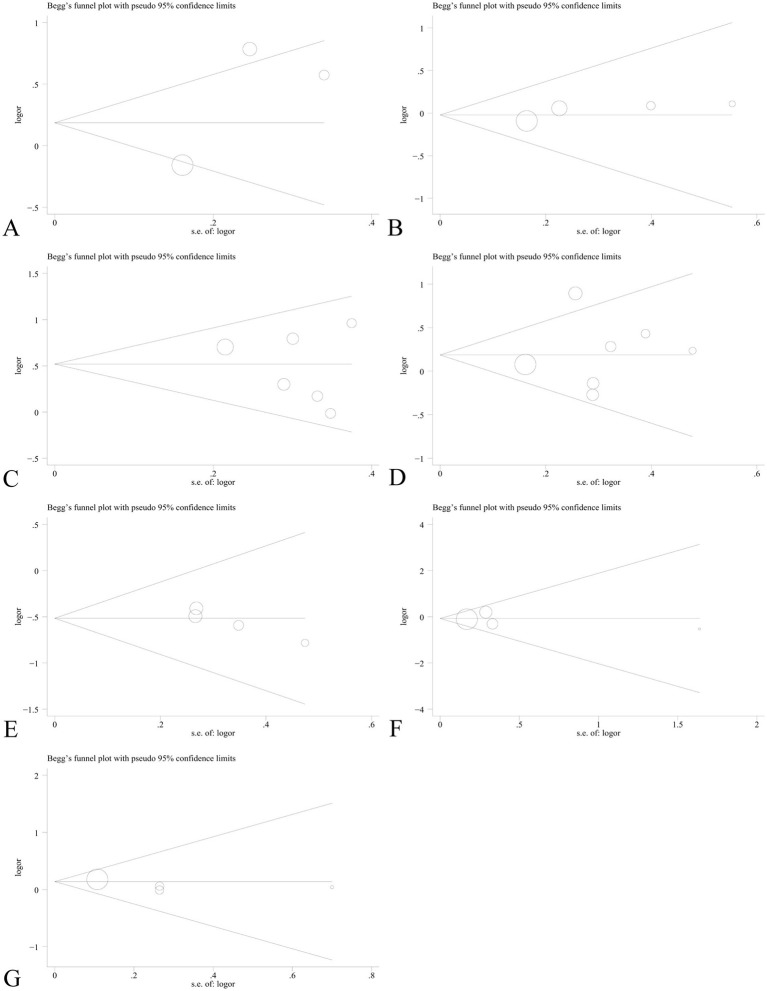
Begg’s funnel plot to detect publication bias using the dominant model. **(A)** IL-1α +4845G/T; **(B)** IL-1β +3953C/T; **(C)** IL-1RA +2018T/C; **(D)** IL-1β -511C/T; **(E)** IL-6 -634C/G; **(F)** IL-6 -174G/C; and **(G)** IL-17A -832A/G.

**Figure 8:**
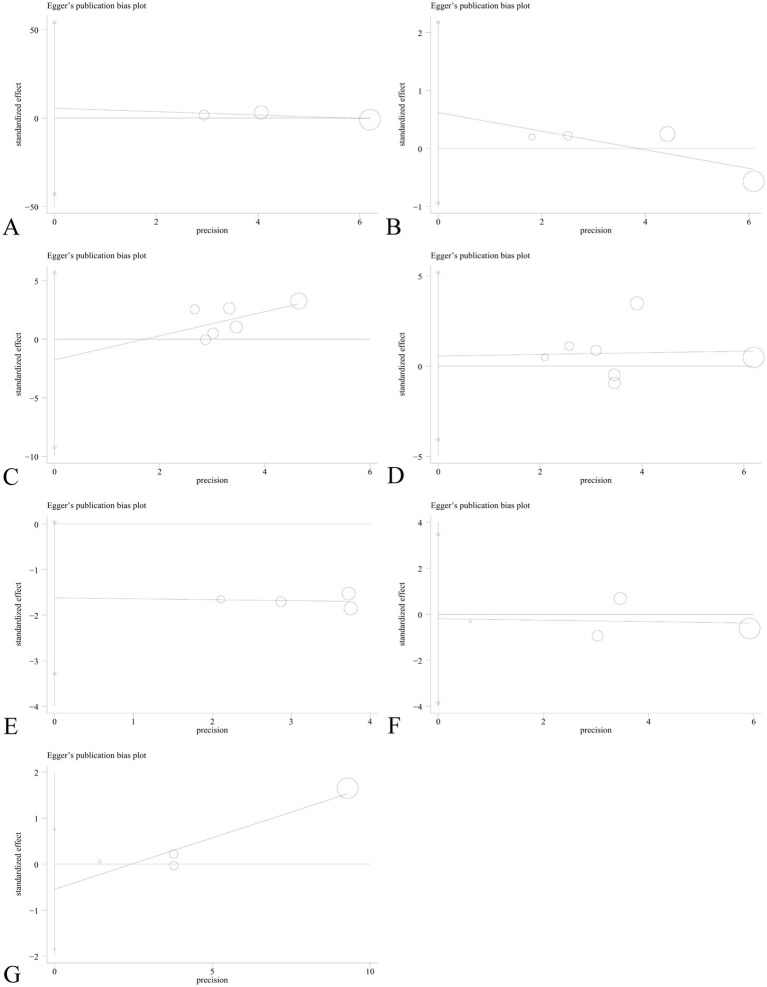
Egger’s linear regression plot to detect publication bias using the dominant model. **(A)** IL-1α +4845G/T; **(B)** IL-1β +3953C/T; **(C)** IL-1RA +2018T/C; **(D)** IL-1β -511C/T; **(E)** IL-6 -634C/G; **(F)** IL-6 -174G/C; and **(G)** IL-17A -832A/G.

Sensitivity analysis was performed to assess the effect of an individual study on the pooled results by omitting each study at a time. The pooled OR values for the correlation between nine IL SNPs and the risk of pneumoconiosis remained consistent, implying that our results were stable and reliable (Figure 6; Table 4). Begg’s funnel plot and Egger’s test were used to estimate the potential publication bias. The symmetrical shapes of funnel plots are shown in Figures 7, 8. As shown in Table 5, except for the allelic, homozygous, heterozygous, and dominant models of the IL-17A -832 gene polymorphism, no statistically significant publication bias was found in the five genetic models of other IL gene polymorphisms.

**Table 4:** Association between multiple IL gene polymorphisms and the risk of pneumoconiosis in the five genetic models excluding studies inconsistent with HWE.

Genetic model		IL-1RA +2018	IL-1β -511	IL-6 -634	IL-6 -174	IL-17A -832
Allele	OR (95%CI)	1.87 (1.49, 2.35)	1.31 (0.90, 1.90)	0.64 (0.50, 0.78)	0.99 (0.81, 1.20)	1.02 (0.89, 1.16)
	*P*	0.000	0.159	0.000	0.897	0.821
	I^2^ (%)	45.6	82.8	0.0	3.7	0.0
	*P_(heterogeneity)_*	0.138	0.000	0.749	0.354	0.821
Homozygote	OR (95%CI)	2.81 (1.57, 5.04)	1.72 (0.84, 3.53)	0.56 (0.35, 0.90)	0.98 (0.67, 1.45)	0.93 (0.67, 1.29)
	*P*	0.001	0.142	0.016	0.937	0.652
	I^2^ (%)	0.0	79.3	0.0	42.1	0.0
	*P_(heterogeneity)_*	0.587	0.001	0.562	0.189	0.974
Heterozygote	OR (95%CI)	1.90 (1.20, 3.00)	1.09 (0.70, 1.69)	0.46 (0.28, 0.76)	1.02 (0.75, 1.38)	1.06 (0.89, 1.27)
	*P*	0.006	0.714	0.002	0.912	0.499
	I^2^ (%)	55.7	67.7	53.5	0.0	0.0
	*P_(heterogeneity)_*	0.080	0.015	0.092	0.396	0.986
Dominant	OR (95%CI)	2.06 (1.35, 3.13)	1.24 (0.75, 2.04)	0.53 (0.40, 0.71)	1.00 (0.75, 1.32)	1.04 (0.88, 1.24)
	*P*	0.000	0.402	0.000	0.990	0.636
	I^2^ (%)	51.5	77.6	2.6	14.2	0.0
	*P_(heterogeneity)_*	0.103	0.001	0.380	0.312	0.996
Recessive	OR (95%CI)	2.40 (1.37, 4.21)	1.65 (0.97, 2.80)	0.73 (0.46, 1.16)	0.96 (0.68, 1.36)	0.95 (0.70, 1.29)
	*P*	0.002	0.064	0.184	0.829	0.755
	I^2^ (%)	0.0	71.3	10.0	0.0	0.0
	*P_(heterogeneity)_*	0.836	0.007	0.343	0.453	0.736

^*^p < 0.05.

**Table 5:** Publication bias for the five genetic models of IL gene polymorphisms.

Variables	Allelic	Homozygous	Heterozygous	Dominant	Recessive
	P _B_ P_E_	P _B_ P_E_	P _B_ P_E_	P _B_ P_E_	P _B_ P_E_
IL-1α +4845	1.000 0.268	1.000 0.157	1.000 0.438	1.000 0.384	1.000 0.159
IL-1β +3953	1.000 0.447	1.000 0.370	1.000 0.264	1.000 0.228	1.000 0.535
IL-1RA +2018	1.000 0.927	0.452 0.184	0.452 0.370	0.707 0.548	0.452 0.163
IL-1β -511	0.368 0.523	0.230 0.141	1.000 0.859	0.764 0.771	0.133 0.048
IL-6 -634	0.308 0.102	0.308 0.250	0.308 0.160	0.308 0.052	0.308 0.248
IL-6 -174	1.000 0.826	1.000 0.960	1.000 0.816	0.734 0.842	1.000 0.888
IL-17A -832	0.089 0.080	0.734 0.049	1.000 0.217	1.000 0.212	0.308 0.035

P _B_, *p*-value of Begg’s rank correlation test; P_E_, *p*-value of Egger’s linear regression test. ^*^p < 0.05.

### 3.6 FPRP results

We investigated the factors influencing FPRP across a range of probabilities to determine whether a specific correlation between IL gene polymorphisms and silicosis warrants attention or is noteworthy. In this context, we discovered that our main results were further supported by FPRP analysis. As shown in Table 6, with a prior probability of <0.25, the IL-1RA +2018 polymorphism was associated with silicosis susceptibility under allelic, homozygous, dominant, and recessive models. Similarly, with a prior probability of 0.25, the allelic, homozygous, heterozygous, and dominant models of the IL-6 -634 polymorphism were correlated with silicosis (*p* < 0.2).

**Table 6:** False-positive report probability analysis of the significant results.

SNP	Genetic model	OR (95% CI)	*P*	Power	Prior probability				
					0.25	0.1	0.01	0.001	0.0001
IL-1α +4845	Allele	1.00 (0.81, 1.22)	0.977	1.000	0.746	0.898	0.990	0.999	1.000
	Homozygote	0.99 (0.60, 1.62)	0.968	1.000	0.744	0.897	0.990	0.999	1.000
	Heterozygote	0.99 (0.77, 1.29)	0.941	1.000	0.738	0.894	0.989	0.999	1.000
	Dominant	1.00 (0.78, 1.24)	0.855	1.000	0.720	0.885	0.988	0.999	1.000
	Recessive	1.00 (0.61, 1.62)	0.984	1.000	0.747	0.899	0.990	0.998	1.000
IL-1β +3953	Allele	1.00 (0.83, 1.19)	0.991	1.000	0.748	0.899	0.990	0.999	1.000
	Homozygote	1.18 (0.74, 1.88)	0.486	0.987	0.596	0.816	0.980	0.998	1.000
	Heterozygote	0.87 (0.68, 1.19)	0.384	1.000	0.535	0.775	0.974	0.997	1.000
	Dominant	0.90 (0.71, 1.14)	0.382	1.000	0.534	0.775	0.974	0.997	1.000
	Recessive	1.36 (0.66, 2.79)	0.402	0.854	0.585	0.809	0.979	0.998	1.000
IL-1RA +2018	Allele	1.60 (1.20, 2.13)	0.002	0.937	0.004^*^	0.012^*^	0.119^*^	0.578	0.932
	Homozygote	2.01 (1.35, 2.99)	0.001	0.490	0.003^*^	0.010^*^	0.103^*^	0.537	0.921
	Heterozygote	1.49 (0.96, 2.32)	0.078	0.904	0.205	0.436	0.895	0.998	0.999
	Dominant	1.65 (1.11, 2.46)	0.014	0.827	0.048^*^	0.132^*^	0.626	0.944	0.994
	Recessive	1.87 (1.28, 2.74)	0.001	0.635	0.006^*^	0.018^*^	0.171^*^	0.675	0.954
IL-1β -511	Allele	1.22 (0.91, 1.64)	0.188	0.999	0.360	0.628	0.949	0.995	0.999
	Homozygote	1.46 (0.86, 2.49)	0.165	0.876	0.361	0.629	0.949	0.995	0.999
	Heterozygote	1.06 (0.75, 1.81)	0.831	0.990	0.716	0.883	0.988	0.999	1.000
	Dominant	1.17 (0.80, 1.72)	0.425	0.997	0.561	0.793	0.977	0.998	1.000
	Recessive	1.42 (0.96, 2.12)	0.086	0.953	0.214	0.449	0.900	0.989	0.999
IL-6 -634	Allele	0.62 (0.51, 0.76)	0.000	0.981	0.000^*^	0.000^*^	0.000^*^	0.004^*^	0.041^*^
	Homozygote	0.57 (0.40, 0.83)	0.003	0.753	0.013^*^	0.039^*^	0.307	0.817	0.978
	Heterozygote	0.50 (0.37, 0.67)	0.000	0.500	0.000^*^	0.000^*^	0.001^*^	0.007^*^	0.065^*^
	Dominant	0.54 (0.42, 0.69)	0.000	0.731	0.000^*^	0.000^*^	0.000^*^	0.001^*^	0.011^*^
	Recessive	0.71 (0.50, 1.01)	0.057	0.974	0.149^*^	0.344	0.852	0.983	0.998
IL-6 -174	Allele	0.95 (0.79, 1.13)	0.562	1.000	0.628	0.835	0.982	0.998	1.000
	Homozygote	1.00 (0.70, 1.44)	0.522	1.000	0.749	0.900	0.990	0.999	1.000
	Heterozygote	0.76 (0.36, 1.60)	0.470	0.865	0.620	0.830	0.982	0.998	1.000
	Dominant	0.82 (0.48, 1.42)	0.479	0.961	0.599	0.818	0.980	0.998	1.000
	Recessive	1.05 (0.76, 1.45)	0.767	1.000	0.697	0.873	0.987	0.999	1.000
IL-17A -832	Allele	1.02 (0.90, 1.15)	0.746	1.000	0.691	0.870	0.987	0.999	1.000
	Homozygote	0.94 (0.69, 1.29)	0.702	1.000	0.678	0.863	0.986	0.999	1.000
	Heterozygote	1.07 (0.90, 1.26)	0.417	1.000	0.556	0.790	0.976	0.998	1.000
	Dominant	1.05 (0.89, 1.23)	0.546	1.000	0.621	0.831	0.982	0.998	1.000
	Recessive	0.96 (0.72, 1.28)	0.781	1.000	0.701	0.875	0.987	0.999	1.000

^*^p < 0.2.

## 4 Discussion

Pneumoconiosis is one of the most crucial occupational diseases worldwide (67). It is characterized by the formation of fibrotic nodular lesions caused by the inhalation of coal and crystalline silica particles that become deposited in the lung parenchyma (68, 69). The pathogenic mechanisms of pneumoconiosis remain poorly explicit. Recently, the incidence of pneumoconiosis has increased markedly, but there is a lack of effective treatment (70–72). The incidence and progression of pneumoconiosis are determined by both the total amount of dust and the intensity of dust exposure (24, 73). Among others, cytokines such as tumor necrosis factor (TNF)-*α*, TGF-*β*, IL-1, IL-4, and IL-6 play crucial roles during the early inflammatory response (29–31). Only a few individuals in the same working environment and exposure period ultimately developed pneumoconiosis, and the severity of disease varied greatly among different individuals (25). It has been reported that genetic factors, involving cytokine gene polymorphisms, could modify the susceptibility of asbestos or silica-related diseases, affecting the progression of disease (30). These differences may be attributed to heritable SNPs contained within regulatory elements of cytokine genes.

The accumulated evidence has shown that IL gene polymorphisms are linked to the risk of pneumoconiosis. In 2001, Yucesoy et al. first reported that TNFa +308, TNFa +238, and IL1RA +2018 were positively correlated with moderate, severe, and overall cases of the disease, respectively (46). Moreover, Wu et al. reported that compared to the IL-17F AA carrier, the GA genotype was strongly associated with the decreased risk of silicosis, implying that the G-allele may serve as a protective factor (31). Similarly, a study proved that the IL-17A -832 and + 7488 SNPs were associated with CWP susceptibility, especially in smokers (49). Another study assessed the association between IL-1β +3953, IL-6 -634, IL-12β +1188 and vascular endothelial growth factor A (VEGFA) rs2010963 and the risk of silicosis and reported that the IL-1β +3953T-allele was significantly related to silicosis susceptibility, which was a hazard factor in coal miners (47). However, the results remain inconclusive and controversial. To accurately evaluate the association between nine common IL SNPs and the risk of pneumoconiosis, we carried out a comprehensive analysis of all relevant studies.

A total of 20 relevant articles covering 35 studies were incorporated in this study to accurately evaluate the association between IL gene SNPs and pneumoconiosis. The pooled results indicated that the IL-1RA +2018 gene polymorphism was significantly associated with the risk of pneumoconiosis. There were remarkable correlations between IL-1RA +2018 and the increased risk of pneumoconiosis among Asians and Caucasians, and the C-allele might serve as a hazard factor in these two populations. In contrast, the IL-6 -634 variant was significantly correlated with the decreased risk of pneumoconiosis among Asians, suggesting that the IL-6 -634G-allele variant might play a protective role among Asians. When the subgroup analysis was performed based on the type of disease, we found positive correlations between the IL-1RA +2018 genotype and the increased risk of silicosis and CWP. Conversely, the IL-6 -634 genotype was significantly associated with the lower risk of silicosis and CWP.

As an important member of cytokines, IL-1 is mainly secreted by mononuclear macrophages and involved in the innate inflammation and acquired immunity. IL-1 gene cluster generally contains three interrelated genes in the 430-kb region that encode the IL-1*α*, IL-1*β* and IL-1RA +2018 proteins (33). Each of these genes contains exonic SNPs that influence their expression by augmenting either message stability or the rate of mRNA synthesis. The local release of IL-1 could facilitate extracellular interstitial component accumulation by enhancing the activity of collagenase (74). Among these, IL-1β could promote the migration of activated phagocytes to the site of inflammation, where they activate fibroblasts and produce other pro-inflammatory cytokines, such as IL-6 and IL-8, thereby amplifying the inflammatory response (75). A study reported that carriers of the IL-1β -511 TT genotype markedly increased the pneumoconiosis risk in the Russian population (47). Another study discovered no obvious association between the IL-1β -511 variant and silicosis in the Chinese population. Studies indicated that the IL-1β-511 variant markedly affects the transcriptional activity only in the context of other IL-1β promoter polymorphisms, namely IL-1β-31 (76).

IL-1RA is an anti-inflammatory protein that competitively intercepts the binding of IL-1 to IL-1 receptor without a transducing signal (77). The increased IL-1RA levels can protect against cytokine-induced lung injury (78). The genetic variant has been reported to affect the IL-1RA/IL-1 ratio and modulate inflammatory processes (79, 80). The frequency of the IL-1RA C-allele was increased in several inflammatory diseases, such as systemic lupus erythematosus, ulcerative colitis, fibrosing alveolitis, and silicosis, which seems to play a crucial role in the development of diseases (46, 81). In agreement with some studies, the IL-1RA +2018 polymorphism was closely related to the overall prevalence of silicosis (23). The gene is characterized by a variable number of tandem repeats of the 86-base pair (VNTR) in intron 2. As a synonymous SNP in exon 2, the IL-1RA +2018 variant was in linkage disequilibrium with VNTR (82). There are three possible protein-binding sites in the region around the VNTR of IL-1RA, such as the IFN-α silencer, IFN-β silencer, and acute-phase response element (83). Studies have shown that the IL-1RA C-allele might have no direct effect on the mRNA expression but may indirectly affect the mRNA expression through its linkage with VNTR (82, 83). The variant may interfere with the binding of transcription factors to these regulatory elements or affect RNA stability. The interaction between IL-1RA +2018 and TNF-α-238 showed a strong independent association between each SNP and silicosis, which may be attributed to the overlapping functions of inflammatory cytokines (23).

Different IL SNPs may affect the progression of pneumoconiosis by some underlying mechanisms. The human IL-6 gene is localized on chromosomes 7p21-24, and certain known loci, including -174 and -634, in its promoter region have been widely studied in pneumoconiosis (84). Studies have shown that the -174G-allele is correlated with a higher expression of plasma IL-6, while the C-allele is correlated with a lower expression of the same. The IL-6 -174 and -634 gene polymorphisms are functionally significant, and they exhibit a weak linkage disequilibrium (39, 40, 85). Our results revealed that the frequencies of the IL-6 -634 G alleles in the case and control groups were 25.7 and 35.2%, respectively, and IL-6 -634 was significantly associated with the risk of pneumoconiosis, compared to IL-6 -174. The IL-6 -634 variant may decrease the pneumoconiosis risk by influencing the expression of IL-6, which provides a biologically plausible description to confirm our results (86, 87). As for the IL-17A A-832 polymorphisms, we did not find any significant associations. Being as a relatively novel cytokine, IL-17 connects adaptive and innate immune responses, playing a role in the pathogenesis of silicosis in different ways (88, 89). Chen et al. reported that the GA genotype of IL-17F +7488 was negatively correlated with silicosis, while the GG + GA genotype of IL-17A +832 weakened the protective role of IL-17F in the Chinese population (48). It has been reported that the IL-17F +7488 G-allele was correlated with the increased risk of accelerated silicosis, compared to IL-17A +832 in the Tunisian population, which might be ascribed to the variance of genotype frequency and the function of IL-17 in different ethnicities (49). The possible mechanism is that the IL-17F +7488 mutation induced a substitution of histidine (His) to arginine (Arg) at the amino acid 161 site, and this conversion makes the mutant IL-17F an antagonist of wild IL-17F, thereby influencing the transcription rate of IL-17 (90, 91).

There were several inherent limitations taken into account in the study. First, there was a relatively small number of individual studies and samples for certain IL SNPs, such as IL-1α +4845G/T, IL-1β +3953C/T, and IL-17A -832A/G, resulting in insufficient statistical power and decrease in the reliability of results. Second, some confounding factors could not be ruled out, including matched age and sex, time and levels of dust exposure between the case and control, and the results might be influenced by unadjusted estimates for raw insufficient data. Third, almost all studies focused on Asians in IL-6 -634C/G and IL-17A -832A/G, and on Caucasians in IL-1α +4845G/T, restricting the generalization of results in other ethnicities. Therefore, further large sample size studies with different ethnic populations are needed to assess these results.

## 5 Conclusion

In conclusion, the results have provided a comprehensive evidence that the IL-1RA +2018T/C and IL-6 -634C/G polymorphisms were correlated with the risk of pneumoconiosis. The IL-1RA +2018 variant remarkably increased the pneumoconiosis risk in Asians and Caucasians, while the IL-6 -634 genotype decreased the pneumoconiosis risk among Asians. The IL-1RA +2018 genotype enhanced the risk of CWP and silicosis. Moreover, the IL-6 -634 G-allele mutant decreased the predisposition to silicosis and CWP risk, respectively. Further large-scale case–control studies should be conducted to investigate the potential association between various IL genes and the etiology of pneumoconiosis. Therefore, this meta-analysis demonstrated that IL gene polymorphisms are significantly associated with pneumoconiosis susceptibility.

## Data Availability

The original contributions presented in the study are included in the article/Supplementary material, further inquiries can be directed to the corresponding authors.
