# Supplementary material for: Association between interleukin gene polymorphisms and the risk of pneumoconiosis: a systematic review and meta-analysis
Source: Front Med (Lausanne). 2025 Mar 20;12:1479730. doi: 10.3389/fmed.2025.1479730 (PMC11967403; doi:10.3389/fmed.2025.1479730)
Supplement: Supplementary file 1 [file Data_Sheet_1.zip › Data Sheet 1 - Version 2/Table S1.doc]

Table 1. Distribution of various IL gene polymorphisms in the pneumoconiosis.

| First author | Year | Genotype distribution | | | | | | HWE |
| --- | --- | --- | --- | --- | --- | --- | --- | --- |
|  |  | Case | | | Control | | | *P*-value |
|  |  | AA | Aa | aa | AA | Aa | aa |  |
| **3 Studies for IL-1α +4845G/T gene polymorphism** | | | | | | | | |
| Yucesoy | 2001 | 224 | 59 | 4 | 125 | 30 | 1 | 0.5771 |
| Ates | 2008 | 44 | 18 | 5 | 62 | 26 | 4 | 0.5523 |
| Yucesoy | 2008 | 162 | 104 | 25 | 179 | 121 | 33 | 0.0671 |
| **4 Studies for IL-1β +3953C/T gene polymorphism** | | | | | | | | |
| Yucesoy | 2001 | 90 | 136 | 27 | 43 | 90 | 5 | 0.0000^*^ |
| Ates | 2008 | 14 | 39 | 14 | 18 | 60 | 14 | 0.0033^*^ |
| Wu F | 2008 | 172 | 11 | 0 | 106 | 4 | 1 | 0.0009^*^ |
| Yucesoy | 2008 | 187 | 93 | 17 | 203 | 110 | 22 | 0.1843 |
| **6 Studies for IL-1RA +2018T/C gene polymorphism** | | | | | | | | |
| Yucesoy | 2001 | 149 | 101 | 24 | 113 | 39 | 5 | 0.4759 |
| Fan XY_a | 2006 | 33 | 27 | 20 | 60 | 43 | 22 | 0.0068 |
| Fan XY_b | 2006 | 24 | 11 | 10 | 60 | 43 | 22 | 0.0068 |
| Wang DJ | 2006 | 38 | 32 | 5 | 100 | 33 | 4 | 0.5313 |
| Ates | 2008 | 25 | 29 | 13 | 35 | 46 | 11 | 0.4837 |
| Wang YW | 2012 | 35 | 29 | 4 | 52 | 15 | 1 | 0.9448 |
| **7 Studies for IL-1β -511C/T gene polymorphism** | | | | | | | | |
| Fan XY_a | 2006 | 20 | 37 | 23 | 38 | 63 | 25 | 0.9039 |
| Fan XY_b | 2006 | 12 | 16 | 17 | 38 | 63 | 25 | 0.9039 |
| Wu F | 2008 | 52 | 89 | 42 | 22 | 58 | 31 | 0.5841 |
| Yucesoy | 2008 | 119 | 137 | 47 | 136 | 161 | 43 | 0.6628 |
| Dang ZC | 2012 | 35 | 29 | 56 | 31 | 29 | 60 | 0.0000^*^ |
| Volobaev | 2018 | 40 | 66 | 23 | 78 | 54 | 6 | 0.3762 |
| Zhou Y | 2022 | 11 | 10 | 24 | 13 | 11 | 21 | 0.0090^*^ |
| **5 Studies for IL-6 -634C/G gene polymorphism** | | | | | | | | |
| Liu_a | 2006 | 44 | 13 | 9 | 33 | 33 | 11 | 0.5585 |
| Liu_b | 2006 | 28 | 7 | 3 | 23 | 16 | 6 | 0.2527 |
| Fan XY | 2007 | 82 | 22 | 16 | 62 | 43 | 15 | 0.0927 |
| Dang ZC | 2013 | 80 | 8 | 32 | 63 | 15 | 42 | 0.0000^*^ |
| Xu XZ | 2022 | 91 | 62 | 7 | 72 | 62 | 16 | 0.6289 |
| **5 Studies for** **IL-6 -174G/C gene polymorphism** | | | | | | | | |
| Zhai | 2001 | 99 | 0 | 0 | 159 | 1 | 0 | 0.9684 |
| Ates | 2008 | 37 | 19 | 11 | 31 | 53 | 8 | 0.0281 |
| Yucesoy | 2008 | 114 | 113 | 45 | 122 | 134 | 58 | 0.0523 |
| Fan XY | 2007 | 120 | 0 | 0 | 120 | 0 | 0 | _ |
| Volobaev | 2018 | 24 | 54 | 24 | 50 | 78 | 34 | 0.7258 |
| **5 Studies for** **IL-17A -832A/G gene polymorphism** | | | | | | | | |
| Chen Y_1 | 2014 | 53 | 51 | 9 | 57 | 50 | 9 | 0.0000 |
| Chen Y_2 | 2015 | 48 | 52 | 6 | 59 | 59 | 8 | 0.1780 |
| Han RH | 2015 | 350 | 293 | 50 | 353 | 279 | 57 | 0.8578 |
| Hassani | 2017 | 4 | 23 | 21 | 5 | 35 | 22 | 0.0821 |
| Zhang Z | 2019 | 101 | 103 | 15 | 116 | 109 | 17 | 0.2027 |

^*^P<0.05.
