# Supplementary material for: Association between interleukin gene polymorphisms and the risk of pneumoconiosis: a systematic review and meta-analysis
Source: Front Med (Lausanne). 2025 Mar 20;12:1479730. doi: 10.3389/fmed.2025.1479730 (PMC11967403; doi:10.3389/fmed.2025.1479730)
Supplement: Supplementary file 1 [file Data_Sheet_1.zip › Data Sheet 1 - Version 2/Table S2.doc]

**Supplementary table 2.** NOS for various IL gene polymorphisms in the risk of pneumoconiosis.

| Author | Year | Selection | | | |  | Comparability |  | Exposure | | | | NOS |
| --- | --- | --- | --- | --- | --- | --- | --- | --- | --- | --- | --- | --- | --- |
|  |  | Adequate definition of case | Representative of the cases | Selection of controls | Definition of controls |  | Comparability of cases and controls on the basis of the design or analysis |  | Ascertainment of exposure | Same method of ascertainment for cases and controls | Non-response rates | |  |
| Yucesoy(46) | 2001 | ☆ | ☆ | ☆ | ☆ |  | ☆ |  | ☆ | ☆ |  | | 7 |
| Zhai(64) | 2001 | ☆ | ☆ |  | ☆ |  | ☆ |  | ☆ | ☆ |  | | 6 |
| Fan XY_a(57) | 2006 | ☆ | ☆ | ☆ | ☆ |  | ☆☆ |  | ☆ | ☆ |  | | 8 |
| Fan XY_b(57) | 2006 | ☆ | ☆ | ☆ | ☆ |  | ☆☆ |  | ☆ | ☆ |  | | 8 |
| Liu_a(60) | 2006 | ☆ | ☆ | ☆ | ☆ |  | ☆ |  | ☆ | ☆ |  | | 7 |
| Liu_b(60) | 2006 | ☆ | ☆ | ☆ | ☆ |  | ☆ |  | ☆ | ☆ |  | | 7 |
| Wang DJ(61) | 2006 | ☆ | ☆ | ☆ | ☆ |  | ☆ |  | ☆ | ☆ |  |  | 7 |
| Ates(59) | 2008 | ☆ | ☆ | ☆ | ☆ |  | ☆ |  | ☆ | ☆ |  | | 7 |
| Wu F(31) | 2008 | ☆ | ☆ | ☆ | ☆ |  | ☆☆ |  | ☆ | ☆ | ☆ | | 9 |
| Yucesoy(63) | 2008 | ☆ | ☆ | ☆ | ☆ |  | ☆ |  | ☆ | ☆ |  | | 7 |
| Fan XY(56) | 2007 | ☆ | ☆ | ☆ | ☆ |  | ☆☆ |  | ☆ | ☆ |  | | 8 |
| Dang ZC(55) | 2012 | ☆ | ☆ |  | ☆ |  | ☆ |  | ☆ | ☆ |  | | 6 |
| Wang YW(23) | 2012 | ☆ | ☆ | ☆ | ☆ |  | ☆☆ |  | ☆ | ☆ |  | | 8 |
| Dang ZC(54) | 2013 | ☆ | ☆ |  | ☆ |  | ☆ |  | ☆ | ☆ |  | | 6 |
| Chen Y_1(48) | 2014 | ☆ | ☆ | ☆ | ☆ |  | ☆ |  | ☆ | ☆ |  | | 7 |
| Chen Y_2(53) | 2015 | ☆ | ☆ | ☆ | ☆ |  | ☆ |  | ☆ | ☆ |  | | 7 |
| Han RH(58) | 2015 | ☆ | ☆ | ☆ | ☆ |  | ☆☆ |  | ☆ | ☆ | ☆ | | 9 |
| Hassani(49) | 2017 | ☆ | ☆ |  | ☆ |  | ☆ |  | ☆ | ☆ |  | | 7 |
| Volobaev(47) | 2018 | ☆ | ☆ | ☆ | ☆ |  | ☆☆ |  | ☆ | ☆ |  | | 8 |
| Zhang Z(65) | 2019 | ☆ | ☆ | ☆ | ☆ |  | ☆ |  | ☆ | ☆ |  | | 7 |
| Zhou Y(66) | 2022 | ☆ | ☆ |  | ☆ |  | ☆☆ |  | ☆ | ☆ |  | | 7 |
| Xu XZ(62) | 2022 | ☆ | ☆ |  | ☆ |  | ☆☆ |  | ☆ | ☆ |  | | 7 |
