# Supplementary material for: Association between interleukin gene polymorphisms and the risk of pneumoconiosis: a systematic review and meta-analysis
Source: Front Med (Lausanne). 2025 Mar 20;12:1479730. doi: 10.3389/fmed.2025.1479730 (PMC11967403; doi:10.3389/fmed.2025.1479730)
Supplement: Supplementary file 1 [file Data_Sheet_1.zip › Data Sheet 1 - Version 2/Table S5. PRISMA 2009 Flow Diagram.doc]

**PRISMA 2009 Flow Diagram**

**Screening**

**Included**

**Eligibility**

**Identification**

Records identified through database searching (n = 1,303): PubMed (n = 139), Embase (n = 337), Web of science (n = 368), Cochrane Library (n = 4), Scopus (n = 295), Google Scholar (n = 26),

CNKI (n = 53), and Wanfang (n = 81).

Additional records identified through other sources
(n = 0)

Records after duplicates removed
(n = 619)

Records screened
(n = 619)

Records excluded

(n = 487)

Full-text articles assessed for eligibility
(n = 132)

Excluded (n = 112)

no genotype and other diseases (n = 76)

other genes and IL SNPs (n = 27)

duplicate and incomplete data (n = 9)

Studies included in qualitative synthesis
(n = 20)

Studies included in quantitative synthesis

(meta-analysis, n = 35)

3 studies for IL-1α +4845; 4 studies for IL-1β +3953;

6 studies for IL-1RA +2018; 7 studies for IL-1β -511;

5 studies for IL-6 -634; 5 studies for IL-6 -174;

5 studies for IL-17A -832.
